# Comprehensive Analysis of the Immune Microenvironment in Checkpoint Inhibitor Pneumonitis

**DOI:** 10.3389/fimmu.2021.818492

**Published:** 2022-01-12

**Authors:** Xinqing Lin, Jiaxi Deng, Haiyi Deng, Yilin Yang, Ni Sun, Maolin Zhou, Yinyin Qin, Xiaohong Xie, Shiyue Li, Nanshan Zhong, Yong Song, Chengzhi Zhou

**Affiliations:** ^1^ The First School of Clinical Medicine, Southern Medical University, Guangzhou, China; ^2^ State Key Laboratory of Respiratory Disease, National Clinical Research Centre for Respiratory Disease, Guangzhou Institute of Respiratory Health, First Affiliated Hospital, Guangzhou Medical University, Guangzhou, China; ^3^ Department of Respiratory and Critical Care Medicine, Jinling Hospital, Nanjing, China

**Keywords:** checkpoint inhibitor pneumonitis, immune infiltration, immune microenvironment, aberrant pathway activation, immune check inhibitor (ICI)

## Abstract

**Background:**

While immune checkpoint inhibitors (ICIs) are a beacon of hope for non-small cell lung cancer (NSCLC) patients, they can also cause adverse events, including checkpoint inhibitor pneumonitis (CIP). Research shows that the inflammatory immune microenvironment plays a vital role in the development of CIP. However, the role of the immune microenvironment (IME) in CIP is still unclear.

**Methods:**

We collected a cohort of NSCLC patients treated with ICIs that included eight individuals with CIP (CIP group) and 29 individuals without CIP (Control group). CIBERSORT and the xCell algorithm were used to evaluate the proportion of immune cells. Gene set enrichment analysis (GSEA) and single-sample GSEA (ssGSEA) were used to evaluate pathway activity. The ridge regression algorithm was used to analyze drug sensitivity.

**Results:**

CIBERSORT showed significantly upregulated memory B cells, CD8+ T cells, and M1 Macrophages in the CIP group. The number of memory resting CD4+ T cells and resting NK cells in the CIP group was also significantly lower than in the Control group. The XCell analysis showed a higher proportion of Class-switched memory B-cells and M1 Macrophages in the CIP group. Pathway analysis showed that the CIP group had high activity in their immune and inflammatory response pathways and low activity in their immune exhaustion related pathway.

**Conclusions:**

In this study, we researched CIP patients who after ICIs treatment developed an inflammatory IME, which is characterized by significantly increased activated immune cells and expression of inflammatory molecules, as well as downregulated immunosuppressive lymphocytes and signaling pathways. The goal was to develop theoretical guidance for clinical guidelines for the treatment of CIP in the future.

## Introduction

Immune checkpoint inhibitors (ICIs) are a beacon of hope for non-small cell lung cancer (NSCLC) patients (1–3). However, the immune system may be activated by ICIs, specifically T-cell immunity, causing it to attack normal tissues and organs. This can result in immunotoxic reactions and ICIs-related adverse events (IRAs), checkpoint inhibitor pneumonitis (CIP) is one of the more common IRAs (4). The incidence of CIP reported in clinical trials is roughly 3% to 5% (5–9). Meta-analysis shows that the total incidence of CIP and severe CIP in lung cancer patients is higher than that of other cancer patients (9, 10). Studies have shown that the total incidence of CIP is approximately 3.1% to 4.1%, while the incidence of severe CIP is 1.4% (9, 10).

At present, the mechanisms underlying CIP are not fully understood, but based on current research, the following are considered viable possibilities: (1) the imbalance of the activity and proportion of T cells caused by an increased number of activated T cells and a decreased number of regulatory T cells (Tregs); (2) the activation of preexisting autoantibodies; (3) increased levels of inflammatory cytokines; and 4) a cytotoxic reaction caused by ectopic expression of CTLA-4. It should be noted that CTLA-4 inhibitor and CTLA-4, which can be expressed by normal pituitary cells, can enhance the inflammatory responset (11).

It is believed that the inflammatory state of the lung and the microenvironment of tumor inflammation that are caused by NSCLC may also be related to the development of CIP (11). After treatment with atezolizumab, the levels of c-reactive protein and IL-6 in NSCLC patients with CIP were reported to have increased in comparison to their baseline levels (12). Various baseline and functional abnormalities of lymphoid and myeloid alveolar cell types in patients with CIP were present as well, which is an abnormality that involves the upregulation of pro-inflammatory molecules and the downregulation of the anti-inflammatory process in T cells and bone marrow cells (4). Despite these statistics, we did not perform any systematic analysis of the immune microenvironment in the CIP group or Control group after ICIs treatment. Instead, we hoped to explore and analyze the characteristics of the IME in CIP patients comprehensively by means of bioinformatics. After fully understanding the manifestation of IME in CIP patients, we hope to provide theoretical guidance for the prevention and treatment of CIP in clinics.

## Methods

### Immunotherapy Cohort Collection

The NSCLC patients treated with ICIs, which we referred to as ICI-NSCLC patients in this study, came from The First Affiliated Hospital, Guangzhou Medical University, Guangzhou, Guangdong, China. This cohort included 8 patients (CIP group) who developed CIP after ICIs treatment and 29 patients who did not developed CIP (Control group) (**Supplementary Table 1**). Surgical biopsies were obtained from these NSCLC patients who received ICIs and were clinically diagnosed by computed tomography (CT) as CIP and non-CIP patients. These samples were finally confirmed as CIP and non-CIP by pathology. Finally, the above samples were made into formalin fixation and paraffin embedding (FFPE) sections. See the “**Supplementary Methods**” section for details on the RNA-seq results of the ICI-NSCLC samples.

### Immune Infiltration Analysis

We used CIBERSORT and xCell analysis to evaluate the immune cell content of ICI-NSCLC patients derived from their RNA-seq data (13, 14). We also collected immune genes to further evaluate the difference in immune gene expression (15, 16) between the CIP and Control group, which we further verified using immunohistochemistry (IHC), as well as with flow cytometry. See the “**Supplementary Methods**” section for more details on IHC and flow cytometry analysis methods.

### Enrichment Analysis

In addition to testing immune cells, we used GSEA to evaluate the expression data of the NSCLC patients (17). From here, we obtained the enrichment scores and p-values of the pathways in each group using the gene ontology biological process (GO-BP), gene ontology cellular component (GO-CC), gene ontology molecular function (GO-MF), the Kyoto Encyclopedia of Genes and Genomes (KEGG), and Reactome pathway analysis (18). Enrichment analysis, or more specifically the enrichGO function in the ClusterProfiler R package, was used to evaluate the fold enrichment score and p-value of differential genes in the GP-BP, GO-CC, GO-MF and KEGG pathways. In addition, the ssGSEA algorithm (19) was used to evaluate the performance of each patient based on their GO-BP, GO-CC, GO-MF, KEGG, and REACTOME pathway data. The gene set of the ssGSEA enrichment analysis was derived from hallmark gene sets, curved gene sets (C2), and ontology gene sets (C5) in the molecular signatures database (MsigDB) (20). Finally, we used the calculate_sig_score function in the IOBR R package to calculate the score of each patient in each immune-related pathway.

### Drug Sensitivity Analysis

Based on the expression data of the NSCLC patients, we used the ridge regression algorithm in the R package pRRophetic (21) function to predict the sensitivity of the NSCLC patients to drugs in the Genomics of Drug Sensitivity in Cancer (GDSC) database (22). Through this analysis, we determined the IC50 value that indicates each patient’s drug sensitivity.

### Statistical Analysis

A Mann-Whitney U test was used to compare the expression of immune related genes, immune scores, and immune cell ratios between the CIP group and the Control group. The edge R package was then used to analyze the differences of expression data between the CIP and Control groups (23). In this differential analysis, P<0.05, |Log2 Fold Change (FC)|>1 was taken as the cut-off of the differential gene. Volcano maps and box diagrams were drawn by the ggplot2 R package, while a heat map was drawn using the Complexheatmap R package (24). The p-value was bilateral and less than 0.05. All the analyses were completed using the R software Version. 3.6.1.

## Results

### Differences in Expression Profiles Between the CIP and Control Group

**Figure 1:**
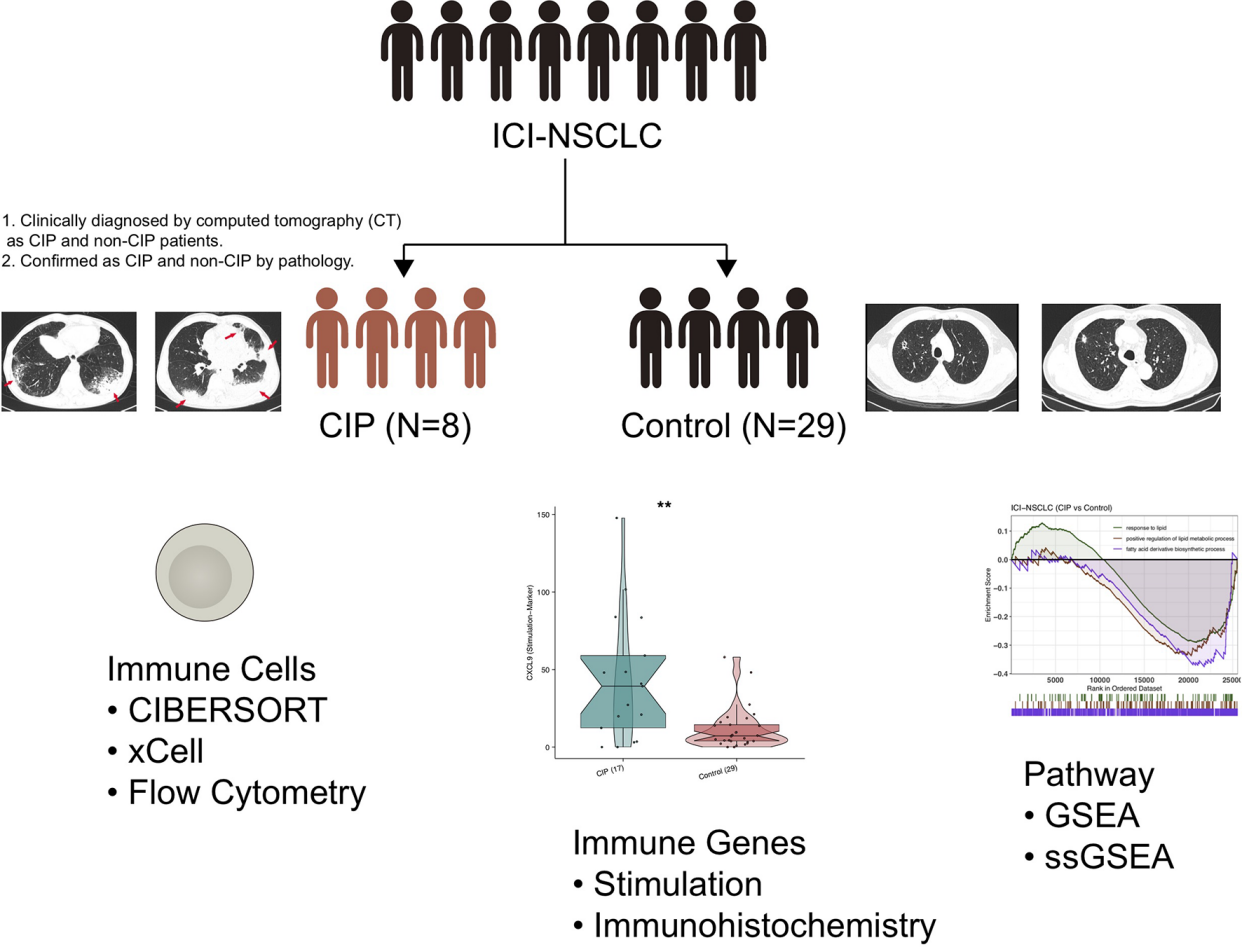
Study design for this research. **P < 0.01.

**Figure 2:**
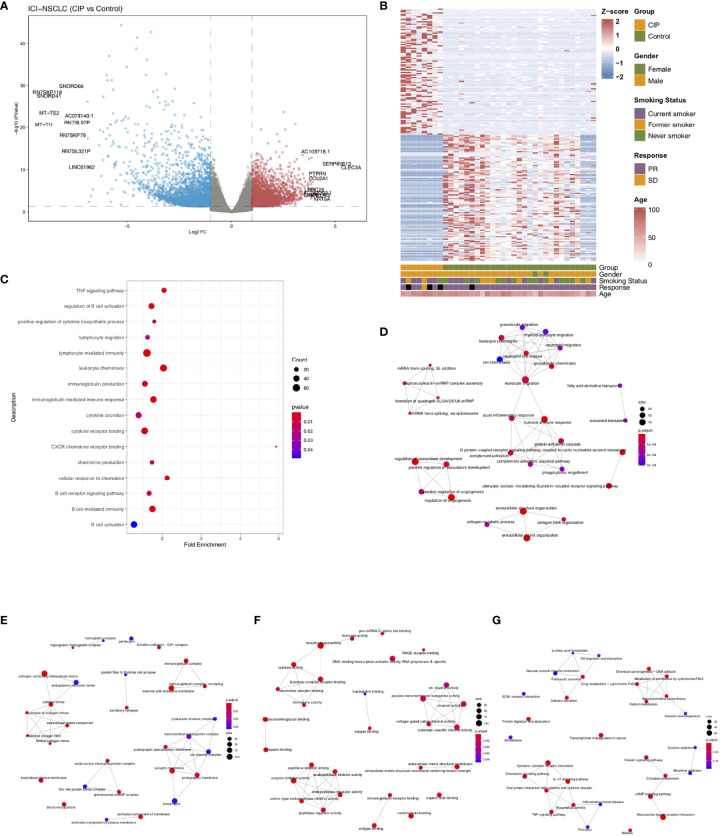
The differences between the CIP and Control group in the expression of NSCLC. **(A)** A volcano plot graph depicting the differences in the expression of NSCLC in each group. The red points represent the significantly upregulated genes. The blue points represent the significantly downregulated genes. The cut-offs we set for p-value and |log2FC| for the differential expression genes were 0.05 and 1, respectively. **(B)** The heatmap depicting the CIP and Control group’s top 100 DEGs in the manifestation of NSCLC. **(C)** The dotplot representing the enrichment pathways in CIP. The emapplot graph representing the relationship between the signaling pathways of GO-BP **(D)**, GO-CC **(E)**, GO-MF **(F)**, and KEGG **(G)**.

To explore the IME of NSCLC patients who developed CIP with ICI-treatment, we collected 8 NSCLC patients who developed CIP after receiving the ICI-treatment and 29 NSCLC patients who were still normal after receiving the same treatment. These two groups of patients were named the CIP group and the Control group. The process of this study is shown in detail in **Figure 1**. First, we analyzed the difference in the expression profiles of the CIP group and the Control group. We selected |log2FC|=1 and p-value = 0.05 as the cut-off for screening the differential genes. We used a volcano map as seen in **Figure 2A** in order to visualize these significantly upregulated or downregulated genes, of which there were 10. In order to further compare the expression differences between the differential genes between the CIP and the Control group, we used a heatmap to display the top 100 genes that showed the most significant difference between the two groups (**Figure 2B**). We also wanted to understand the differences between the pathway activities represented by the differential genes. **Figure 2C** shows the significantly different pathways enriched by the differential genes in both groups as revealed through enrichment analysis. Examples of this include B cell activation, cytokine secretion, immunoglobulin production, chemokine production, lymphocyte mediated immunity, lymphocyte migration, regulation of B cell activation, B cell receptor signaling pathway enrichment, positive regulation of cytokine biosynthetic process, cytokine receptor binding, B cell-mediated immunity, immunoglobulin-mediated immune response, TNF signaling pathway enrichment, and cellular response to chemokine. The activation scores of leucocyte chemotaxis and CXCR chemokine receptor binding were significantly higher in the CIP group than in the Control group (**Figure 2C**). The pathway network diagrams in **Figures 2D–G** show that immune-related pathways and inflammatory-related pathways both play a mediating role between other major pathways (**Figures 2D–G**).

### Differences in Immune Pathway Activity Between the CIP and Control Groups

**Figure 3:**
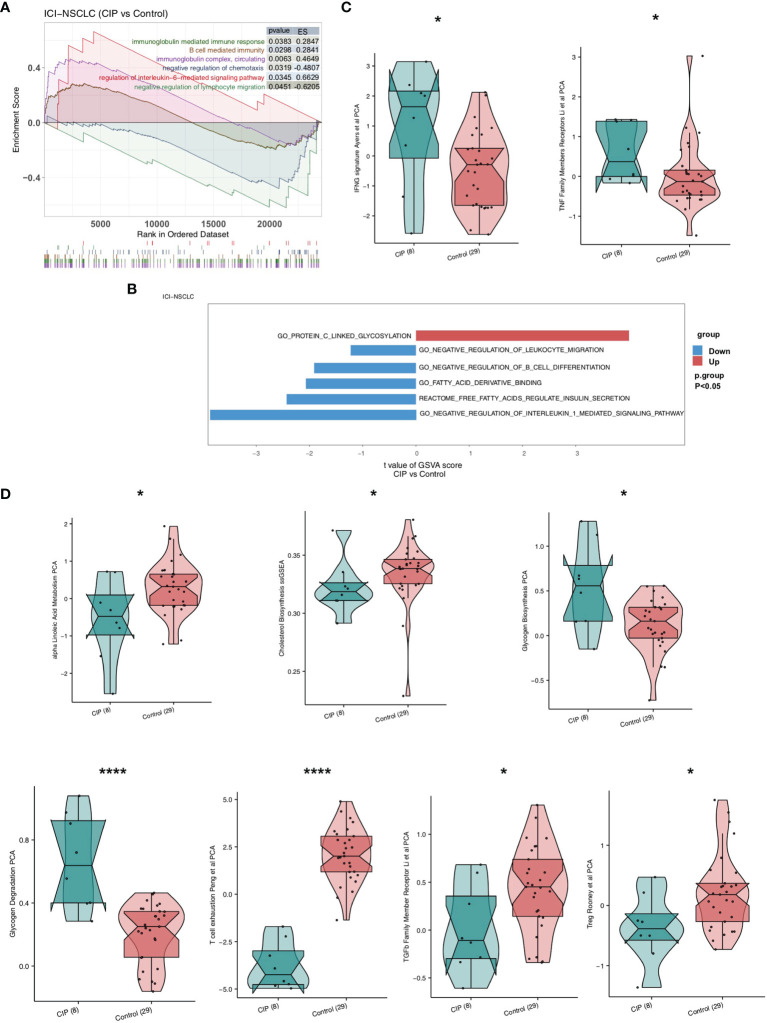
A comparison between the activity of the signaling pathways in NSCLC of the CIP and Control group. **(A)** The significantly upregulated activation due to immune signaling in the CIP group shown in comparison to the Control group based on the GSEA results. **(B)** A comparison of the ssGSEA scores of the CIP and Control groups’ NSCLC manifestations. **(C)** A comparison of activation due to immune signaling in both groups as estimated by the ssGSEA results. **(D)** A comparison of the immune-exhaustion signaling in both groups as estimated by the ssGSEA results. *P < 0.05; ****P < 0.0001.

In order to explore the difference between the immune pathway activity of the CIP and Control group, we used the GSEA and ssGSEA algorithm to evaluate the proportion of immune cells in each NSCLC patient. The results of GSEA showed that the enrichment degree of immune-related or inflammatory pathway in the CIP group was significantly higher than of that of the Control group (**Figure 3A**). This high enrichment degree manifested in significant upregulation of the interleukin-6-mediated signaling pathway, B cell-mediated immunity, and immune response mediated by circulating immunoglobulin complex. The negative regulation of chemotaxis and lymphocyte migration was significantly decreased in the CIP group in comparison to the Control group. We used the ssGSEA algorithm to calculate the pathway score of each NSCLC patient and the Bayesian Limma test to analyze the difference in pathway activity between the CIP group and the Control group (**Figure 3B**). The results showed that the ssGSEA score of GO_PROTEIN_C_LINKED_GLYCOSYLATION in the CIP group was significantly higher than that of the Control group. Conversely, the ssGSEA scores of GO_NEGATIVE_REGULATION_OF_INTERLEUKIN_1_MEDIATED_SIGNALING_PATHWAY,REACTOME_FREE_FATTY_ACIDS_REGULATE_INSULIN_SECRETION,GO_FATTY_ACID_DERIVATIVE_BINDING,GO_NEGATIVE_REGULATION_OF_B_CELL_DIFFERENTIATION, and GO_NEGATIVE_REGULATION_OF_LEUKOCYTE_MIGRATION in the CIP group were significantly lower than those of the Control group. After calculating the immune-related pathways of the NSCLC patients using the IOBR R package, we found the IFNG signature defined by Ayers et al. using principal component analysis (PCA). By basing our application of the PCA on Li et al.’s previous study as seen in **Figure 3C**, we found that the level of TNF receptors in the CIP group was significantly higher than in the Control group (P < 0.05). However, after using Treg Rooney et al.’s application of PCA as seen in **Figure 3D**, we found that the Treg activity of the CIP group was significantly lower than that of the Control group (P < 0.05). Still, in some immune depletion pathways, such as the ssGSEA score of Treg Rooney et al. PCA, the transforming growth factor (TGF-β) family member receptor analysis using the Li et al. PCA, the T cell exhaustion analysis using the Peng et al. PCA, the alpha linoleic acid metabolism PCA, and the cholesterol Biosynthesis in the CIP group showed significantly lower scores than in the Control group (P < 0.05; **Figure 3D**). The activity of glycogenesis PCA and glycogenesis degradation PCA in the CIP group was significantly higher than in the Control group (P < 0.05; **Figure 3D**).

### Differences Between the Immune Cells and Inflammatory Molecules in the CIP and Control Group

**Figure 4:**
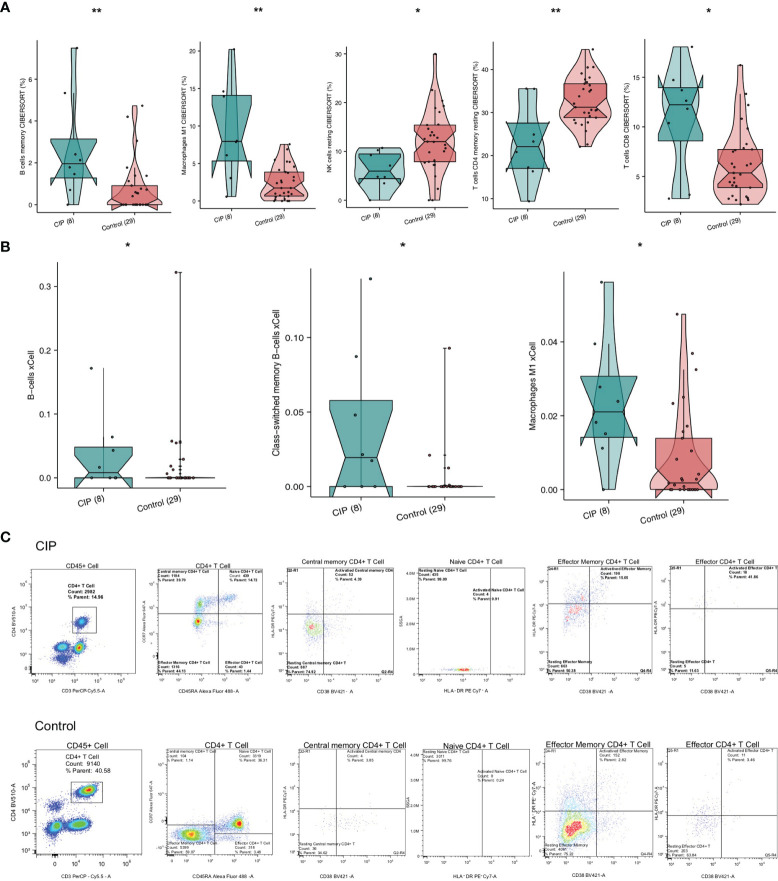
A comparison of immune cells and inflammatory genes in the CIP and Control groups’ manifestations of NSCLC. **(A)** A comparison of the proportion of the immune cells found in each group’s NSCLC based on the CIBERSORT results. **(B)** A comparison of the scores of the immune cells of each group’s NSCLC based on the xCell algorithm results. **(C)** After utilizing flow cytometry analysis, we found low infiltration of the resting effector memory CD4+ T cells in the CIP group, and high infiltration of activated effector CD4+ T cells in the CIP group. *P < 0.05; **P < 0.01.

In order to explore the differences in the immune cell ratio of each group of NSCLC patients, we used CIBERSORT and the xCell algorithm. **Figure 4A** shows that memory B cells, CD8+ T cells, and M1 macrophages were significantly upregulated in the CIP group. On the contrary, the quantity of memory resting CD4+ T cells and resting NK cells in the CIP group was significantly lower than in the Control group (all P < 0.05). The XCell algorithm analysis results showed that there was a higher proportion of class-switched memory B cells and M1 macrophages in the CIP group (**Figure 4B**). We also utilized flow cytometry analysis (**Figure 4C** and **Supplementary Table 2**), which revealed that CIP patients had fewer memory CD4+T cells than Control group patients with proportions of 50.38% to 70.22%, respectively. However, it should also be noted that the CIP patients had more activated effector CD4+T cells than the Control group patients with proportions of 41.86% to 3.46%, respectively.

**Figure 5:**
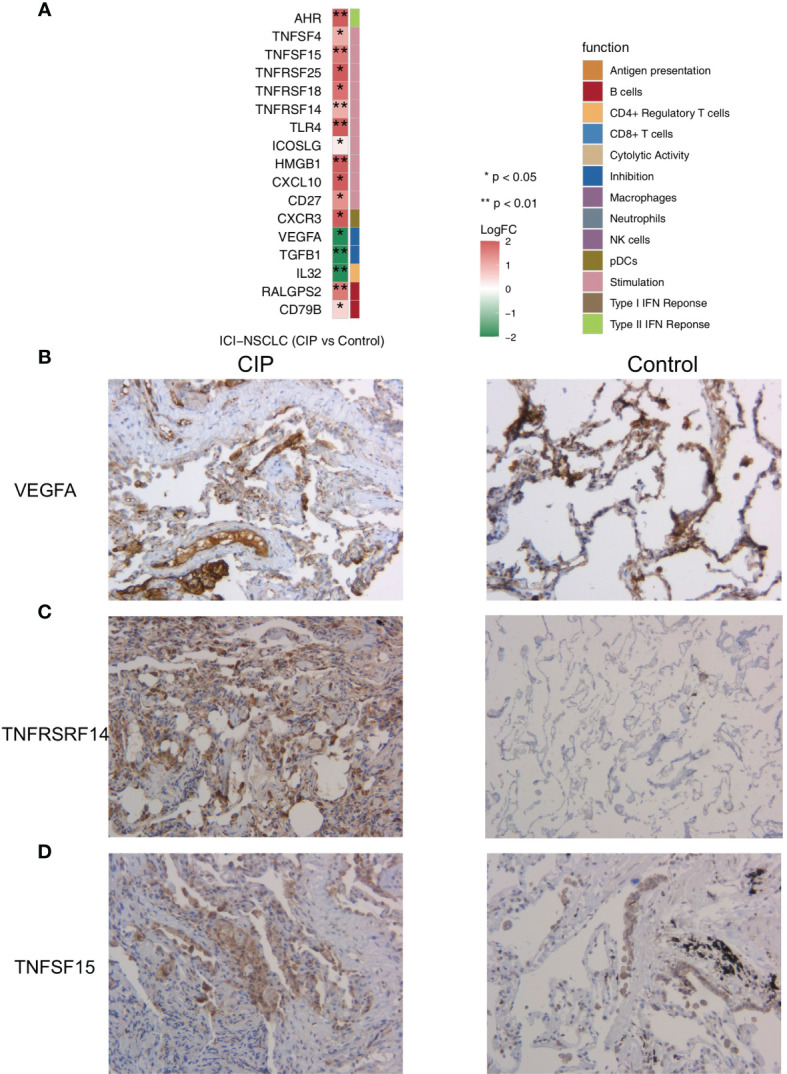
**(A)** A comparison of the expression of the immune-related genes in the CIP and Control groups’ NSCLC. **(B)** Immunohistochemistry analysis (VEGFA) of the CIP and Control group. **(C)** Immunohistochemistry analysis (TNFRSF14) of the CIP and Control group. **(D)** Immunohistochemistry analysis (TNFSF15) of the CIP and Control group.

In order to further explore the differences in the gene expression of immune-related functions between the CIP and Control groups, we obtained a list of immune-related genes from a recently published study. We found that in the CIP group, there was a significant increase in the expression level of CD79B and RALGPS2 (P < 0.05; **Figure 5A**), while the expression of marker-IL32 in CIP CD4+Tregs was lower than that in the Control group (P < 0.05; **Figure 5A**). The expression of some immune depletion molecules, such as TGFB1 and vascular endothelial growth factor (VEGFA), in the CIP group were significantly lower than that in the Control group (P < 0.05; **Figure 5A**), while the CIP group showed lower expression of TNFSF4, TLR4, CD27, TNFRSF14, ICOSLG, CXCL10, TNFSF15, TNFRSF18, and HMGB1. The expression of genes represented by inflammatory molecules such as TNFRSF25 and AHR was significantly higher in in the CIP group (**Figure 5A**). The immunohistochemical results showed that the CIP group had lower VEGFA expression with a ratio of 80% to 40%, higher TNFRSF14 expression with a ratio of 60% to 10%, and higher TNFSF15 expression with a ratio of 30% to 10% (**Figures 5B–D**).

### Differences in the Drug Sensitivity of CIP and Control Group Patients

**Figure 6:**
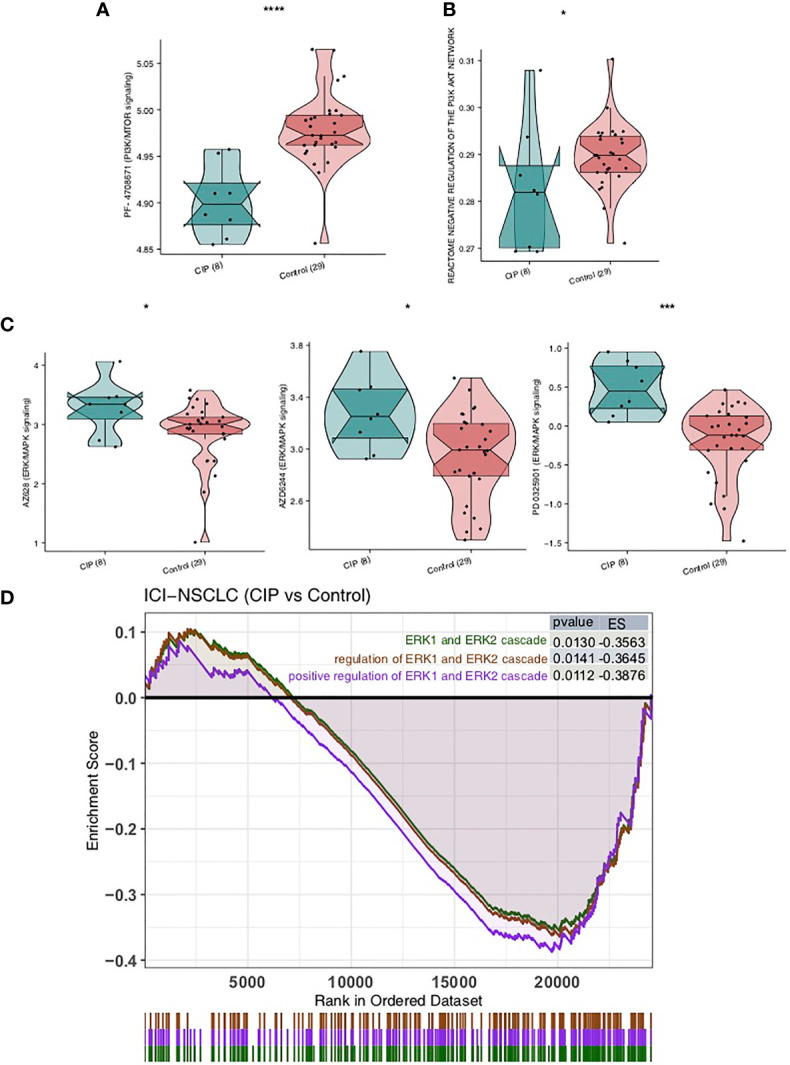
A comparison of drug sensitivity of NSCLC patients in both the CIP and Control groups. **(A)** A comparison of the groups’ IC50 values of PI3K-AKT signaling inhibitors. **(B)** A comparison between the ssGSEA scores of the PI3K-AKT signaling in each group’s NSCLC. **(C)** A comparison between the IC50 values of the ERK/MAPK signaling inhibitors in each group’s NSCLC. **(D)** Based on the GSEA results, the CIP group displayed significantly downregulated ERK/MAPK signaling in comparison to the Control group. *P < 0.05; ***P < 0.001; ****P < 0.0001.

We calculated the sensitivity of each patient to 138 drugs in the GDSC database by using the expression data on NSCLC and the ridge regression algorithm, then compared the IC50 value of the CIP group and Control group using the Mann-Whitney U test. We found that the IC50 value of inhibitors in the PI3K/MTOR signaling pathway, AZD6482, and PF-4708671 in the CIP group was significantly lower than in the Control group, as seen in **Figure 6A** (all P < 0.05). This indicates that these two drugs may be used in combination for CIP treatment. We also used ssGSEA to further explore the activity of PI3K-AKT signaling and found that the activity of the PI3K-AKT pathway in the CIP group was significantly higher than that in the Control group (**Figure 6B**). In addition, we found that IC50 values of inhibitors of the ERK/MAPK signaling pathway, AZ628, AZD6244, and PD0325901, were significantly higher in the CIP group than in the Control group, as seen in **Figure 6C** (all P < 0.05). Finally, we used GSEA to further explore the activity of ERK/MAPK signaling and found that the activity of the ERK/MAPK signaling pathway in the CIP group was significantly lower than that in the Control group (**Figure 6D**).

## Discussion

**Figure 7:**
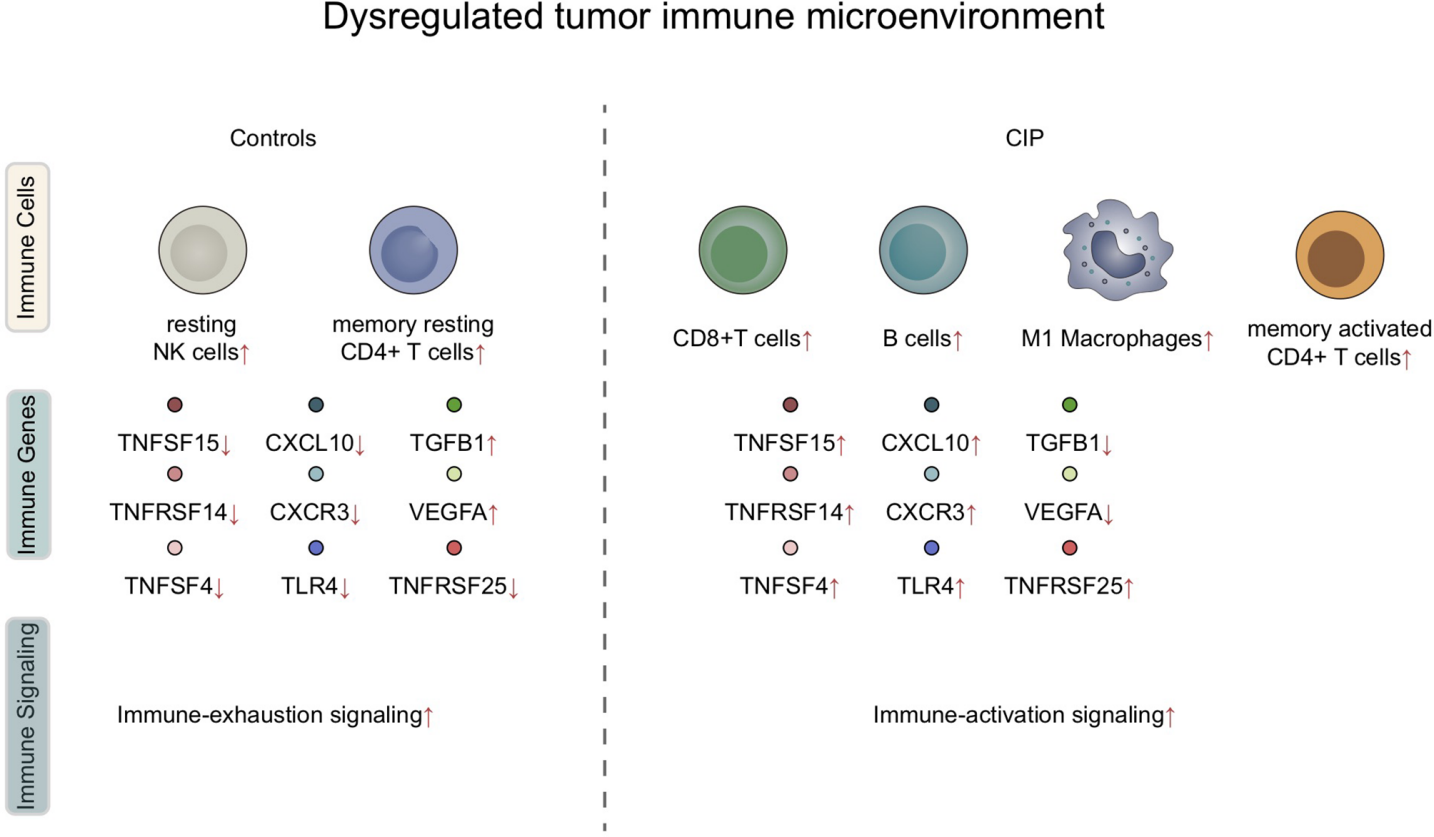
A summary of the dysregulated immune microenvironment in CIP.

In this study, we found that significant differences were present in the IME of each subject group after receiving immune checkpoint treatment for NSCLC. The reaction of the immune microenvironment in the CIP group was characterized by inflammatory IME, which included significantly upregulated activated lymphocytes, as well as highly enriched inflammatory and immune response-related pathways. At the same IME, in the IME of the CIP group individuals, the proportion of some immunosuppressive cells, as well as the activities of some immunosuppressive pathways, showed a significant downward trend (**Figure 7**).

Based on these findings, the inflammatory immune microenvironment may play a vital role in the occurrence and development of CIP by stimulating the significant upregulation of activated lymphocytes and the expression of inflammatory molecules, as well as significant activation of immune signaling and inflammatory-related pathways. In a recent study, Suresh et al. (4) found that bronchoalveolar lavage (BAL) samples from CIP patients displayed lymphocyte proliferation and were predominantly composed of CD4+T cells. They also observed that the number of central memory T cells (Tcm) increased while the expression of CTLA-4 and PD-1 decreased. Studies have shown that PD-1+ and CTLA-4+Tregs have negative regulatory effects on the pro-inflammatory response of CD8+T cells, Tcms, and macrophages (25, 26). In addition, a subset of CD4+ T cells in CIP tends to have high rates of IFN-γ and TNF-α production (4). This high expression of inflammatory molecules and immune cells plays an important role in the development of CIP. It is widely known that the transient expression of IL-1β can induce lung inflammation, increase TNF-α, and contribute to progressive tissue fibrosis (27). The CXCR3/CXCL9-11 axis plays a key role in promoting the entrance of Th1 cells, CD8+T cells, and NK cells into the IME, thus producing a T cell inflammatory IME which has a strong anti-tumor effect (28). IFN-γ activates antigen-presenting cells (APC), such as macrophages and DC, after which APC secretes a large amount of CXCL9. This in turn causes the transfer of a large amount of CXCR3+CD8+T and CXCR3+CD4+T cells into the tumors, which take part in anti-tumor activities (29). In this study, the CIP group displayed significantly increased levels of activated memory CD4+T cells, B cells, CD4+TCM, M1 macrophages, and class-switched memory B cells, while it also displayed significantly decreased levels of M2 macrophages and other static immune cells. In addition, in the TNF family molecules, the expression of CXCL10 in the CIP group was significantly higher than in the Control group. These results suggest that significantly enriched activated lymphocytes and significantly upregulated inflammatory molecules may be one of the mechanisms of CIP manifestation and development.

Exhaustion or significant downregulation of immunosuppressive pathway activity may also be involved in the occurrence and development of CIP. As important pathways in the IME, mitochondrial reactive oxygen species (ROS), glycolysis, and lipid metabolism play a key role in immunosuppression and immune depletion. High ROS content in the IME can inhibit the activation and proliferation of T cells and other anti-tumor functions, as the activation of T cells is necessary in order to stimulate T-cell receptors (TCRs) by inducing signal transduction pathways and transcription factors. When Ca2+ flows into CD4+T cells through TCR-dependent activities, it results in the production of mitochondrial ROS and inhibits the activation of CD4+T cells (30, 31). In another study, Kunisada et al. (32) used metformin, a specific mitochondrial antioxidant, to maintain Treg in a high glycolytic state and reduce the level of mitochondrial ROS. The immunosuppressive activity of Treg was decreased while the immune function of T cells was enhanced. Brown et al. (33) showed that increasing the production of ROS can lead to apoptosis of CD4+T cells and promote the formation of hepatocellular carcinoma. In addition, glycolysis and lipid metabolism also play an important role in the activation and depletion of T cells. The activated neutrophils and M1 macrophages also rely mainly on glycolytic pathway for energy supply. Treg cells and M2 macrophages mainly rely on oxidative phosphorylation of fatty acid β to provide energy (34–36). Zhang et al. (37) used cell experiments to show that the lipid metabolism pattern of M2 macrophages changed when activated by tumor cells, which depend on fatty acid oxidation (FAO) to obtain energy. Furthermore, after ROSs and NLRP3 inflammatory corpuscles were activated, the secretion of IL-1β was promoted, and the rates of proliferation, migration, and invasion of hepatoma cells were accelerated. Wu et al. (38) found that unsaturated fatty acids derived from lipid droplets in tumor cells induced polarization of myeloid cells with M2 macrophages by promoting mitochondrial respiration. Activated T cells also increased the intake of fatty acids, but inhibited FAO while promoting lipid synthesis (39). In this study, the CIP group had significantly downregulated immune depletion or immunosuppressive pathway activity, which resulted in significantly downregulated ROS and fat metabolism, as well as significantly upregulated glycolysis activity. These results suggest that significantly downregulated immune depletion may be another major mechanism of CIP manifestation and development.

## Conclusions

In this study, we explored data on NSCLC patients who developed CIP after ICIs treatment and exhibited an inflammatory immune microenvironment, which is mainly characterized by significantly increased activated immune cells, significantly increased expression of inflammatory molecules, and downregulated immunosuppressive lymphocytes and signal pathways, in the hopes of providing theoretical guidance to clinical guidelines for the prevention and treatment of CIP in the future.

## Data Availability Statement

The data presented in the study are deposited in the data.mendeley.com, a public and community-supported repository, as follows: https://data.mendeley.com/datasets/8c3x28r5hk/. Accession number: Lin, Xinqing (2021), “Comprehensive Analysis of the Immune Microenvironment in Checkpoint Inhibitor Pneumonitis”, Mendeley Data, V1, doi: 10.17632/8c3x28r5hk.1.

## Ethics Statement

The studies involving human participants were reviewed and approved by First Affiliated Hospital of Guangzhou Medical University (No.2020-95). The patients/participants provided their written informed consent to participate in this study. Written informed consent was obtained from the individual(s) for the publication of any potentially identifiable images or data included in this article.

## Author Contributions

Conceptualization, YS and CZ. Formal analysis, XL. Visualization, XL. Writing–original draft, JD, HD, YY, NS, MZ, YQ, XX, SL, and NZ. Writing–review & editing, XL, JD, HD, YY, NS, MZ, YQ, XX, SL, and NZ. All authors read and approved the final manuscript.

## Funding

This study was supported by grants from the State Key Laboratory of Respiratory Disease-The Independent project (SKLRD-Z-202206), Fundamental and Applied Fundamental Research Project of City-School (Institute) Joint Funding Project, Guangzhou Science and Technology Bureau (202102010357), Wu Jieping Medical Foundation (320.6750.2020-19-8) and State Key Laboratory of Respiratory Disease-The open project [SKLRD-OP-202111].

## Conflict of Interest

The authors declare that the research was conducted in the absence of any commercial or financial relationships that could be construed as a potential conflict of interest.

## Publisher’s Note

All claims expressed in this article are solely those of the authors and do not necessarily represent those of their affiliated organizations, or those of the publisher, the editors and the reviewers. Any product that may be evaluated in this article, or claim that may be made by its manufacturer, is not guaranteed or endorsed by the publisher.

## Supplementary Material

Supplementary Table 1 The clinical characteristics of the NSCLC of the CIP and Control group.

Supplementary Table 2 The result of the Flow cytometry.
