# Supplementary material for: Comprehensive Analysis of the Immune Microenvironment in Checkpoint Inhibitor Pneumonitis
Source: Front Immunol. 2022 Jan 12;12:818492. doi: 10.3389/fimmu.2021.818492 (PMC8790088; doi:10.3389/fimmu.2021.818492)
Supplement: Supplementary file 1 [file DataSheet_1.docx]

**Supplementary methods**

**NSCLC patient tissue processing, gene sequencing and data processing**

**1. RNA extractions and RNA sequencing**

For RNA extractions, tissue sections were first lysed and homogenized with the TissueLyser (Qiagen). Subsequent RNA extractions were performed with the Qiagen RNeasy Mini Kit according to the instructions provided by the manufacturer. The RNA quality was assessed with a Bioanalyzer 2100 DNA Chip 7500 (Agilent Technologies), and samples with an RNA integrity number (RIN) of over 7 were further analyzed by RNA-seq. All sequencing reactions were performed on an Illumina HiSeq 2000 instrument (Illumina, San Diego, CA, USA). RNA-seq was performed with RNA extracted from FFPE samples (n=37). cDNA libraries were prepared from poly(A)-selected RNA by applying the Illumina TruSeq protocol for mRNA. The libraries were then sequenced with a 2 x 100 bp paired-end protocol.

**2. RNA-seq Data processing**

We used HISAT2 (version 2.1.0)(1) with the default setting to map the RNA-seq data to the human reference genome (NCBI38/hg38). We aggregated the read counts at the gene level using HTSeq(2).

**3. Immunohistochemistry**

Whole tissue sections from paraffin embedded samples were immunostained using undiluted hybridoma supernatant of anti-TNFRSF14 (2G6-2C7, Novus, H00008764-M01), anti-TNFSF15 (Novus, MAB74422-sp), and anti-VEGFA (Novus, ab215715). The immunohistochemical staining was performed according to manufacturer’s instructions.

**4. Flow cytometry**

Flow cytometry was performed using a Northern Light 3000 flow cytometer (Cytek Biosciences). The instrument was configured with 3 lasers (405 nm, 488 nm, 640 nm) and 38 detectors. The 38 detectors are spread out across the entirety of the emission spectrum, which facilitates deconvolution of fluorescent signals and allows for simultaneous use of dyes that are incompatible on a traditional flow cytometer with a single detector, e.g. APC and Alexa Fluor 647. Dyes were selected with distinct spectral properties to allow for accurate marker detection. For each staining, 1 × 106 PBMCs were used. Pretitrated volumes of antibodies were prepared as a master mix and mixed with PBMCs in the tubes. The final volume in each tube was 100 μl and incubated for 40 min at room temperature in dark. Then, the cells were washed by PBS twice. Subsequently, the cells were resuspended in stain buffer (FBS) for acquisition in Cytek Northern Light 3000 flow cytometer (3-lase :405 nm, 488 nm, 640 nm) using the SpectroFlo Software v3.0.0.1. Compensation was calculated using SpectroFlo software (Cytek) and data was exported in .fcs file format.

**Reference**

1. Kim D, Langmead B, Salzberg SL. HISAT: a fast spliced aligner with low memory requirements. *Nature methods* (2015) **12**:357–360. doi:10.1038/nmeth.3317

2. Anders S, Pyl PT, Huber W. HTSeq--a Python framework to work with high-throughput sequencing data. *Bioinformatics (Oxford, England)* (2015) **31**:166–169. doi:10.1093/bioinformatics/btu638
