# Supplementary material for: Comprehensive Analysis of the Immune Microenvironment in Checkpoint Inhibitor Pneumonitis
Source: Front Immunol. 2022 Jan 12;12:818492. doi: 10.3389/fimmu.2021.818492 (PMC8790088; doi:10.3389/fimmu.2021.818492)
Supplement: Supplementary Table 1 — The clinical characteristics of the NSCLC of the CIP and Control group. [file Table_1.pdf]

	CIP (N=8)	Control (N=29)	Overall (N=37)	
**Gender**				
Male	8 (100%)	27 (93.1%)	35 (94.6%)	P value=0.0823
Female	0 (0%)	2 (6.9%)	2 (5.4%)	
**Age**				
Mean (SD)	67.5 (6.30)	61.3 (9.94)	62.7 (9.55)	
Median [Min, Max]	68.0 [56.0, 75.0]	62.0 [37.0, 77.0]	63.0 [37.0, 77.0]	P value=1
**Smoking_Status**				
Current smoker	5 (62.5%)	9 (31.0%)	14 (37.8%)	
Former smoker	2 (25.0%)	7 (24.1%)	9 (24.3%)	P value=0.2196
Never smoker	1 (12.5%)	13 (44.8%)	14 (37.8%)	
**Response**				
PR	4 (50.0%)	28 (96.6%)	32 (86.5%)	
SD	1 (12.5%)	0 (0%)	1 (2.7%)	P value=0.1515
Missing	3 (37.5%)	1 (3.4%)	4 (10.8%)	
**Sample_Location**				
inferior lobe of left lung	2 (25.0%)	4 (13.8%)	6 (16.2%)	
inferior lobe of right lung	2 (25.0%)	1 (3.4%)	3 (8.1%)	
lung	1 (12.5%)	0 (0%)	1 (2.7%)	
right middle lobe	1 (12.5%)	2 (6.9%)	3 (8.1%)	
superior lobe of right lung	2 (25.0%)	11 (37.9%)	13 (35.1%)	P value=0.1652
left upper lobe	0 (0%)	5 (17.2%)	5 (13.5%)	
right lung	0 (0%)	1 (3.4%)	1 (2.7%)	
right middle and lower	0 (0%)	4 (13.8%)	4 (10.8%)	
upper lobe from the middle lobe of the right lung	0 (0%)	1 (3.4%)	1 (2.7%)	
