# Supplementary material for: Comprehensive Analysis of the Immune Microenvironment in Checkpoint Inhibitor Pneumonitis
Source: Front Immunol. 2022 Jan 12;12:818492. doi: 10.3389/fimmu.2021.818492 (PMC8790088; doi:10.3389/fimmu.2021.818492)
Supplement: Supplementary Table 2 — The result of the Flow cytometry. [file Table_2.pdf]

Cell subtype / Sample	CIP	Control
CD45+ Cell (%)	2.37%	10.91%
CD4+ T Cell	14.96%	40.58%
Central memory CD4+ T Cell	39.70%	1.14%
Naive CD4+ T Cell	14.72%	36.31%
Effector Memory CD4+ T Cell	44.13%	59.07%
Effector CD4+ T Cell	1.44%	3.48%
Activatied Central memory CD4+ T Cell	4.39%	3.85%
Resting Central Memory CD4+ T Cell	74.92%	34.62%
Resting Naive CD4+ T Cell	99.09%	99.76%
Activated Naive CD4+ T Cell	0.91%	0.24%
Activatived Effector Memory CD4+ T	15.05%	2.82%
Resting Effectort Memory CD4+ T Cell	50.38%	75.22%
Activated Effector CD4+ T	41.86%	3.46%
Resting Effector CD4+ T	11.63%	63.84%
